# Uptake of oxLDL and IL-10 Production by Macrophages Requires PAFR and CD36 Recruitment into the Same Lipid Rafts

**DOI:** 10.1371/journal.pone.0076893

**Published:** 2013-10-09

**Authors:** Francisco J. O. Rios, Matheus Ferracini, Mateus Pecenin, Marianna M. Koga, Yajuan Wang, Daniel F. J. Ketelhuth, S. Jancar

**Affiliations:** 1 Department of Immunology, Institute of Biomedical Sciences, University of Sao Paulo, Sao Paulo, Brazil; 2 BHF-Glasgow Cardiovascular Research Centre, Institute of Cardiovascular and Medical Sciences, University of Glasgow, Glasgow, United Kingdom; 3 Center for Molecular Medicine, Department of Medicine, Karolinska Institutet, Stockholm, Sweden; University of Padova, Italy

## Abstract

Macrophage interaction with oxidized low-density lipoprotein (oxLDL) leads to its differentiation into foam cells and cytokine production, contributing to atherosclerosis development. In a previous study, we showed that CD36 and the receptor for platelet-activating factor (PAFR) are required for oxLDL to activate gene transcription for cytokines and CD36. Here, we investigated the localization and physical interaction of CD36 and PAFR in macrophages stimulated with oxLDL. We found that blocking CD36 or PAFR decreases oxLDL uptake and IL-10 production. OxLDL induces IL-10 mRNA expression only in HEK293T expressing both receptors (PAFR and CD36). OxLDL does not induce IL-12 production. The lipid rafts disruption by treatment with βCD reduces the oxLDL uptake and IL-10 production. OxLDL induces co-immunoprecipitation of PAFR and CD36 with the constitutive raft protein flotillin-1, and colocalization with the lipid raft-marker GM1-ganglioside. Finally, we found colocalization of PAFR and CD36 in macrophages from human atherosclerotic plaques. Our results show that oxLDL induces the recruitment of PAFR and CD36 into the same lipid rafts, which is important for oxLDL uptake and IL-10 production. This study provided new insights into how oxLDL interact with macrophages and contributing to atherosclerosis development.

## Introduction

The oxidation of Low Density Lipoprotein (LDL) is a major pathogenic factor in the development of atherosclerosis. Oxidized LDL (oxLDL) accumulates in the sub-endothelial space of the arterial wall and activates endothelial cells, smooth muscle cells and macrophages. The resulting chronic inflammatory response contributes to atherosclerotic plaque progression[1]. Although is well accepted that the uptake of oxLDL has pathophysiologic relevance in atherosclerosis development, the molecular mechanisms of its interaction with macrophages are not fully understood. 

CD36 is one of the main scavenger receptors involved in the uptake of oxLDL by macrophages and has generally been viewed as essential for foam cell formation[2]. Its deficiency greatly reduced the uptake of oxLDL and atherosclerotic lesions in mice models[3,4]. Monocytes from individuals lacking CD36, or *in vitro* experiments using functional blockage of this receptor with antibodies, decreased the oxLDL uptake by 50% [3]. However, other studies have found that a deficiency of CD36 did not prevent macrophage foam cell formation *in vivo*[5,6]. These contradictory observations suggest that additional receptor(s) might be involved in macrophage activation by oxLDL and foam cell formation.

The oxidative modification of LDL generates several oxidized phospholipids (oxPL) that share a common unsaturated fatty acid in the *sn*-2 position of the phosphatidylcholine and confer the ability to bind to CD36[7]. These structural characteristics are also responsible for the interaction of some oxPL with the receptor for Platelet Activating Factor (PAFR) [8], which its expression has been found in macrophages from human atherosclerotic plaques[9]. In previous studies we demonstrated that deficiency or antagonism of PAFR reduced the oxLDL uptake by human macrophages and CD36 expression. It was also found that co-stimulation of CD36 and PAFR is required for IL-8 and MCP-1 production by macrophages exposed to oxLDL[10,11]. We also showed that oxLDL stimulates a number of signal transduction pathways through PAFR engagement including MAPK and PI3K/Akt pathway activation, which leads to the transcription of genes for cytokines and CD36 as well[11]. Because CD36 has a very short cytoplasmic domain[12], it has been suggested that its association with other receptors is required for activating intracellular signaling pathways[13,14].

The PAFR has, in its amino acid sequence, a binding motif for caveolin-1[15], which is a constitutive protein in lipid raft platforms. Thus, we hypothesized that the association of PAFR with CD36 might occur in lipid raft domains. In the present study, we found that oxLDL induces the formation of a complex comprising CD36, PAFR and Flotillin-1 within lipid raft platforms and this is required for the uptake of oxLDL and IL-10 production by macrophages. Moreover, we found colocalization of PAFR and CD36 in human atherosclerotic plaques. 

## Materials and Methods

### Purification and oxidation of LDL

The study was approved by the ethics committee of the Institute of Biomedical Sciences, University of Sao Paulo and the participants provide their written consent. Blood was collected from normolipidemic volunteers and plasma was obtained after centrifugation at 1,000 *g*, 4°C, for 15 min in the presence of EDTA 1 mg/mL. Thereafter, we added benzamidine (2 mmol/L), gentamicin (0.5%), chloramphenicol (0.25%), PMSF (phenyl-methyl-sulfonyl-fluoride) (0.5 mmol/L), and aprotinin (0.1 U/mL) (all acquired from Sigma-Aldrich, St. Louis, MO, USA). LDL (density: 1.019-1.063 g/mL) was isolated by sequential ultracentrifugation (100,000 *g*, 4°C), using a P90AT-0132 rotor (CP70MX ultracentrifuge; Hitachi Koki Co., Ltd, Tokyo, Japan), dialyzed (4°C) against PBS, pH 7.4 containing 1 mmol/L EDTA, filtered (0.22 μm) and stored at 4°C. The protein concentration was determined by the BCA kit (Thermo Scientific, Rockford, IL, USA). Part of the LDL was dialyzed overnight against EDTA-free PBS. The LDL with minimal degree of oxidation (moxLDL) was obtained by dialysis of LDL (2 mg/mL) against 2 μM FeSO_4_· 7H_2_O in PBS (pH 7.2) for 48 h at room temperature in the dark. Highly oxidized LDL (oxLDL) was obtained by incubation of LDL with CuSO_4_ (5 μmol/L per mg of LDL protein/ 18 h/ 37°C). The oxidation for both methods was stopped by the addition of 1 mmol/L EDTA, the degree of oxidation was determined by measuring the concentration of tiobarbituric acid-reactive substances (TBARS) and conjugated dienes. TBARS values for LDL, moxLDL and oxLDL were 0.2, 7.5, and 53.1 nmol/mg protein respectively. Conjugated dienes were mesured by optical density at 234 nm: 2.84, 3.02, and 4.00 for LDL, moxLDL and oxLDL respectively. 

### Cell culture

6–8 week old male C57BL/6 mice were obtained from our own animal facilities and were housed in a room with 12 h light–dark cycle with water and food *ad libitum*. Animal care and research protocols were in accordance with the principles and guidelines adopted by the Brazilian College of Animal Experimentation (COBEA) and approved by the Biomedical Sciences Institute/USP—Ethical Committee for Animal Research (CEEA). We did not perform *in vivo* studies. Bone marrow-derived macrophages (BMDM) were established as previously described by Davies and Gordon (2005)[16], with minor modifications. In brief, femurs were flushed with PBS, using a 26 x 1.2”-gauge needle. Cells were grown in L-Cell conditioned medium (DMEM containing 20% L929 cell-conditioned medium, 15% FCS, 2 mmol/L l-glutamine, 100 U/ml penicillin G, and 100 mg/ml streptomycin) (Gibco, Long Island, NY, USA) , incubated at 37°C in 5% CO_2_. At day 3, new fresh L-Cell conditioned medium was added. Monolayer of macrophages was scrapped at day 6. Macrophages were culture in DMEM/1% for one day before the experiments. 

The monocytic cell line THP-1 was cultured in RPMI-1640 medium supplemented with 5% FCS, 100 U/mL penicillin, 100 μg/mL streptomycin, 2 mM L-glutamine, 15 mM HEPES and 11 mM sodium bicarbonate. Cell cultures were maintained in a humidified atmosphere containing 5% CO_2_ at 37°C. The differentiation of THP-1 monocytes into macrophages was induced by 150 nM phorbol 12-myristate-13-acetate (PMA) for 24 h. Non-adherent cells were removed by aspiration of the supernatant followed by replacement with fresh medium. 

### Uptake of oxLDL

LDL was labeled with Fluorescein isothiocyanate (FITC) (Merck Chemicals, Nottingham, UK), as described previously[17,18]. Breafly, 2 mg/mL of LDL were dialyzed in carbonate buffer (NaHCO_3_ 0.5 mol/L; EDTA 1 mmol/L pH 9.5) containing 2 mg of Fluorescein isothiocyanate (FITC), overnight at 4°C. The unbound FITC was removed by dialysis in PBS/EDTA and PD10 column (Amersham Pharmacia Biotech, Uppsala, Sweden). The FITC concentration in LDL was determined by spectroscopy against FITC standard solution at 495 nm. The F/P (fluorochrome/protein) molar ratio was calculated and admitted in the range of 2.4 to 3 as previously described [19]. FITC-LDL was oxidized by CuSO_4_ (5 μmol/L per mg of LDL protein; 18 h; 37°C). Cells were treated with PAFR antagonists “WEB2170 (50 µmol/L) (Boehringer Ingelheim, Pharma KG, Biberach, Germany), CV3988 (10 µmol/L) (Tocris Bioscience, Bristol, UK)” alone or in combination with anti-CD36 blocking antibody (1 µg/mL) (monoclonal IgA anti-CD36, clone CRF D-2712, BD Biosciences, Franklin Lakes, NJ, USA), for 30 min, then incubated with FITC-oxLDL (30 µg/mL) for 1 h at 37°C. In some experiments the cells were pre-treated with the Methyl-β-cyclodextrin (βCD) or with the inactive analogue α-ciclodextrin (αCD) (all from Sigma-Aldrich, St. Louis, MO, USA) before uptake assay. Cells were washed with cold PBS and fixed with 2% formaldehyde. The uptake of FITC-oxLDL was visualized by fluorescent microscopy and evaluated in flow cytometer (FACS Canto II - Becton BD Biosciences) and the data were analyzed by the software Summit® V4.3 (DakoCytomation). 

### Co-immunoprecipitation and immunoblotting

Macrophages were treated with oxLDL (30 µg/mL) or PAF (10^-7^ mol/L) for 20 min. Resting and activated cells were lysed without agitation on ice for 30 min using HEPES buffer containing 1 mmol/L CaCl_2_, 1 mmol/L MgCl_2_, 1% Triton-x-100, protease inhibitor cocktail (Sigma-Aldrich, St. Louis, MO, USA) and phosphatase inhibitors (Calbiochem- Merck Chemicals, Nottingham, UK). Post-nuclear lysates were incubated overnight with the primary antibody of interest (rabbit anti-PAFR or mouse IgA anti-CD36). Protein A-Sepharose (GE Healthcare, NJ, USA) and protein G-Sepharose (Amersham Pharmacia Biotech, Uppsala, Sweden) were added to samples containing anti-PAFR and anti-CD36, respectively, and incubated for 3 h at 4°C with gentle agitation. Immune complexes bound to beads were washed three times with HEPES buffer containing protease and phosphatase inhibitors, without triton-x-100, and boiled in SDS sample buffer for 5 minutes. Proteins were separated by 10% SDS-PAGE, transferred to a Hybond™ nitrocellulose membrane (GE Healthcare, NJ, USA), and incubated with rabbit-anti-PAFR (Cayman Chemical, Ann Arbor, Michigan, USA) or mouse IgA-anti-CD36 (BD Biosciences, Franklin Lakes, NJ, USA) or with mouse anti-flotillin-1(BD Biosciences, Franklin Lakes, NJ, USA). As secondary antibodies we used anti-rabbit IgG-HPR (1:2,000), anti-mouse-HRP (1:1,000) (Cell Signaling Technology, Beverly, MA, USA), biotin-anti-IgA (1:500) (BD Biosciences, Franklin Lakes, NJ, USA) with streptavidin-HRP (1:200) (Life Technologies, Carlsbad, CA, USA) and visualized using SuperSignal West Pico Chemiluminescent Substrate (Thermo Scientific, Rockford, IL, USA). The resulting autoradiograms were analyzed with the AlphaEaseFC^TM^ software V3.2 beta (Alpha Innotech).

### Transfection of HEK 293T cells

HEK 293T cells (ATCC) were cultured in DMEM supplemented with 10% FCS, 15 mM HEPES, 2 mmol/L L-glutamine, 100 U/mL penicillin and 100 μg/mL streptomycin. The plasmids CD36-pcDNA3.2/V5-DEST, pcDNA3-kozac-mycPAFR and the control plasmid pcDNA3 were purified using a Qiagen Plasmid Kit (Qiagen Inc., Valencia, CA, USA). The day before the transfection, HEK 293T cells were seeded onto 24-well plates at a density of 2 x 10^5^ cells/well. The cells were transiently transfected with 2 µg of total plasmid DNA per well using Lipofectin® Transfection Reagent (Invitrogen-Life Technologies, Carlsbad, CA, USA), according to the manufacturer’s instructions. At 48 h after transfection, the cells were used for the experiments. 

### mRNA expression

RNA was isolated using TRIzol reagents (Life Technologies, Carlsbad, CA, USA). For the real-time reverse-transcriptase polymerase chain reaction (PCR), cDNA was synthesized using the RevertAidTM First Strand cDNA Synthesis Kit (Fermentas Life Sciences, Ontario, USA), according to the manufacturer's instructions. PCR-master mix (Power SyBr® Green, Applied Biosystems, Warrington, UK) containing the specific primers was then added. Primers used were: hIL-10 forward: GATCCAGTTTTACCTGGAGGAG and reverse: CCTGAGGGTCTTCAGGTTCTC; hIL12p40 forward TGCCCATTGAGGTCATGGTG and reverse: CTTGGGTGGGTCAGGTTTGA, and GAPDH forward: GAGTCAACGGATTTGGTCGT and reverse: TTGATTTTGGAGGGATCTCG. Real-time PCR was performed using a Stratagene Mx3005PTM QPCR System (Santa Clara, CA, USA). Relative gene expression was calculated by the 2^-Delta Delta C(T)^ method, as previously described[20]. Data are shown in fold increase related to untreated cells.

### MTT assay

The mitochondrial-dependent reduction of methylthiazolyldiphenyl-tetrazolium bromide (MTT) (Sigma-Aldrich, St. Louis, MO, USA) to formazan insoluble crystals was used to evaluate cell viability. Briefly, 10 µL of 5 mg/ml of MTT in PBS were added to the cells after the treatments. After incubation at 37°C for 2 h, 100 µL of 10% SDS in 0.01 mol/L HCl was added to dissolve the crystals and incubated for 16 h. The absorbance was measured in a Dynatech microplate reader at 570 nm. 

### Confocal microscopy of macrophages

Macrophages (2 x 10^5^) were plated in glass cover slips and treated with oxLDL (30 µg/mL) or PAF (10^-7^ mol/L) (Cayman Chemical, Ann Arbor, Michigan, USA) for 20 minutes, and then washed with PBS. Cells were fixed with 3% paraformaldehyde and blocked with 1% BSA in PBS, before incubation with primary antibodies anti-PAFR (1:100) (Cayman Chemical, Ann Arbor, Michigan, USA) and IgA anti-CD36 (1:100) (BD Biosciences, Franklin Lakes, NJ, USA). FITC Donkey anti-rabbit IgG (1:100) (BioLegend, San Diego, CA, USA), Alexa-647 donkey anti-rabbit (1:200) (Invitrogen-Life Technologies, Carlsbad, CA, USA) or Biotin-anti–mouse IgA (1:200) with streptavidin-PE (1:200) (BD Biosciences, Franklin Lakes, NJ, USA) was used as the secondary antibody. Cells stained with secondary antibody and control antibody were used to control for the background from each fluorophore. Slides were mounted in Prolong® Gold anti-fade reagent with DAPI (4,6-diamidino-2-phenylindole) (Invitrogen-Life Technologies, Carlsbad, CA, USA). Cells were imaged on a Zeiss LSM 510 confocal microscope using 100x oil objective at a 60-fold magnification. Images were analyzed by Pearson’s coefficient in JACoP (Just Another Colocalization Plugin) using the package ImageJ 1.44p (Wayne Rasband, NIH, USA) available in http://imagej.nih.gov/ij. Confocal images were taken with identical settings to allow comparison of staining. Single confocal sections of the cells were captured in multitrack. Each set of frames from a given treatment condition depicts a representative from at least 20 analyzed cells in three independent experiments.

### Confocal microscopy of atherosclerotic plaque tissue

Carotid artery plaques were collected from patients who were undergoing endarterectomy. Informed written consent was obtained from all subjects. The investigation was approved by the Ethical Committee of Northern Stockholm and was in agreement with institutional guidelines and the principles that were set forth in the Declaration of Helsinki. Acetone-fixed sections were incubated with 5% horse serum followed by a polyclonal rabbit anti-human PAF receptor IgG antibody at a concentration of 25 µg/mL. Binding was detected with DyLight 594 anti rabbit IgG antibody (Vector Laboratories, Peterborough, UK). The binding specificity was detected using the PAF receptor blocking peptide (Cayman Chemical, Ann Arbor, Michigan, USA) prior to incubation with the PAF receptor IgG antibody. For double staining of the PAFR and CD36 or CD68, a mouse anti-human CD36 (Cayman Chemical, Ann Arbor, Michigan, USA) or a mouse anti-human CD68 (BD Biosciences, Franklin Lakes, NJ) was used as the primary antibody, followed by DyLight 488 anti-mouse IgG (Vector Laboratories, Peterborough, UK). Lipid autofluorescence was blocked with 0.03% Sudan black B (Sigma Aldrich, USA) in 70% ethanol. Nuclei were visualized with DAPI (Sigma Aldrich, St. Louis, MO, USA), and images were captured with a Leica TCS SP5 confocal microscope. 

### Cytokine measurements

IL-10 (mouse and human), mIL-12p40, and hTGFβ concentration in the supernatants of macrophages were measured using BD OptEIA^TM^ ELISA Set (BD Biosciences, San Diego, CA, USA). 

### Statistical analysis

Data are presented as mean ± SEM. Analysis of variance (ANOVA) and the Student-Newman-Keuls post-test were used to evaluate the statistical significance of the differences between three or more groups. Two-tailed unpaired Student's t-test was used when differences between two groups were analyzed. Significance was assumed if p < 0.05.

## Results

### Uptake of oxLDL and IL-10 production by macrophages requires engagement of CD36 and PAFR

**Figure 1:**
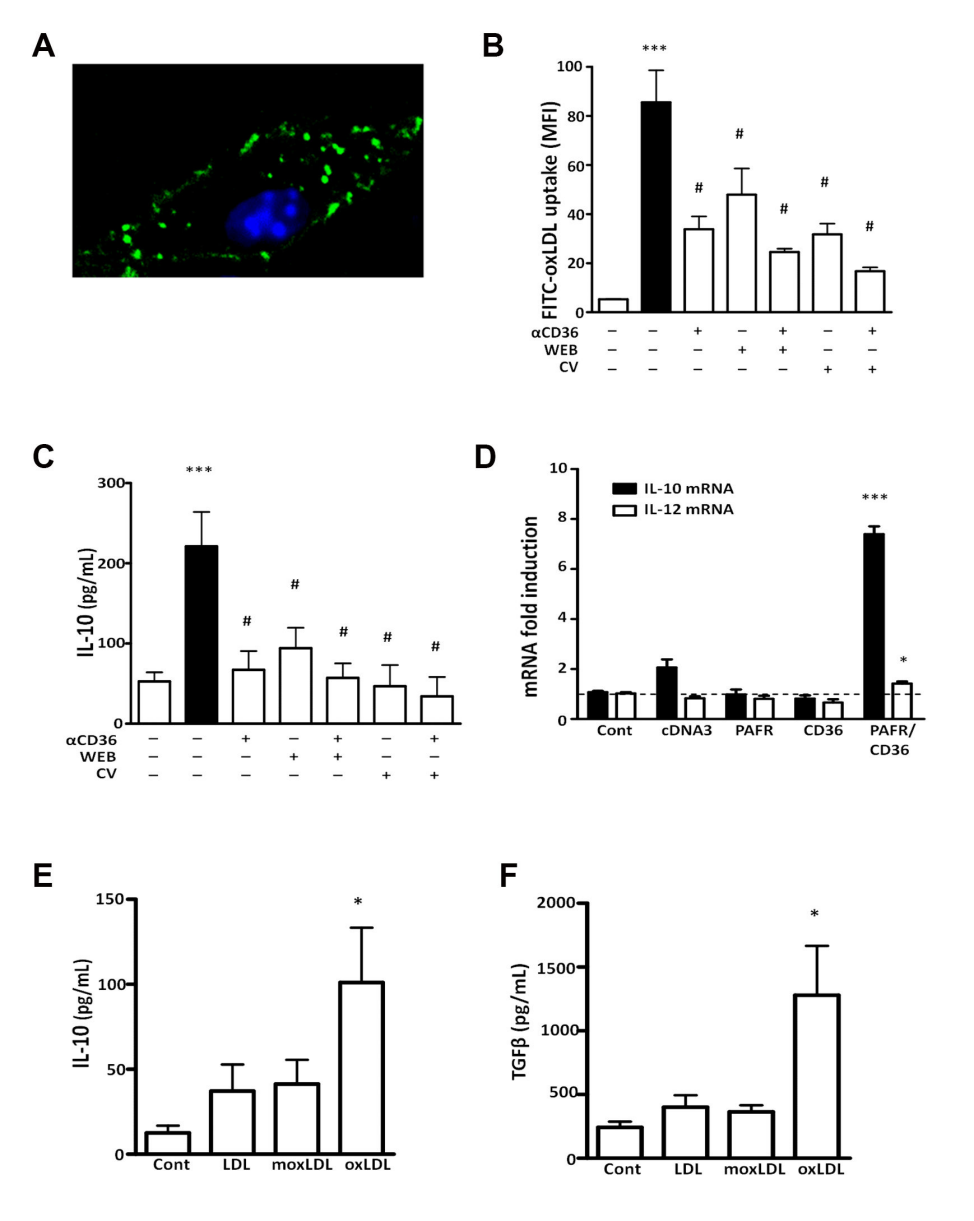
CD36 and PAFR cooperatively mediate the uptake of oxLDL and IL-10 production. Macrophages were treated with mAb to CD36 (1:500) alone or in combination with WEB2170 (50 µmol/L) or CV3988 (10 μmol/L) for 30 min and then incubated with FITC-oxLDL for 1 h. In (A-B) The uptake was visualized in confocal microscopy and measured by FACS (B). In (C), after treatment, cells were stimulated with oxLDL (30 µg/mL) and the IL-10 production was evaluated after 24 h by ELISA. Data are presented as mean ± SEM of the mean fluorescence Intensity (MFI). ***p<0.001 comparing with non-stimulated cells. # p<0.05 comparing cells treated with non-treated cells. In (D) HEK 293T cells were transiently transfected with hCD36 and/or hPAFR and stimulated with oxLDL (30 µg/mL). The mRNA expression for IL-10 and IL-12 was evaluated after 5 h. *** p<0.001; * p<0.05 comparing with cDNA3 plasmid control transfected cells. In (E-F), THP-1 monocytes were differentiated into macrophages with PMA, followed by treatment with LDL, moxLDL or oxLDL (30 µg/mL) for 24 h. IL-10 and TGFβ were measured in the supernatant were measured by ELISA. *p<0.05 comparing with non-stimulated cells.

 Bone marrow-derived macrophages (BMDM) were treated with the blocking antibody to CD36 alone or in combination with two molecularly unrelated PAFR antagonists CV3988 (10 µmol/L) or WEB2170 (50 µmol/L) for 30 min and then incubated with FITC-oxLDL for 1 h. The concentrations of PAFR antagonists were based on previous results from our group[10]. We found a rapid increase in macrophage fluorescence within 30 minutes of FITC-oxLDL addition, which accumulates in the plasma membrane and also in intracellular vesicles (illustrated in Figure 1A). The fluorescence intensity was quantified as a measure of oxLDL uptake. Figure 1B shows that the uptake was reduced by pre-treatment of macrophages with antibodies to CD36 (62% inhibition compared to untreated group). Pre-treatment with PAFR antagonists (WEB2170 or CV3988) also reduced the oxLDL uptake (42% and 61% inhibition, respectively). The combination treatment with anti-CD36 and WEB2170 or anti-CD36 and CV3988 further reduced the oxLDL uptake (71% and 79% inhibition, respectively). PAFR antagonists or anti-CD36 did not affect the cell viability, measured by MTT assay, which was around 98% in all groups. These data show that the uptake of oxLDL by BMDM is higher when both receptors are functional. 

Next, we investigated if PAFR and CD36 are required for IL-10 and IL-12 production, which are cytokines involved with macrophage suppression or activation, respectively. BMDM were pre-treated with the anti-CD36 blocking antibody alone, or in combination with the PAFR antagonists 30 min before stimulation with oxLDL (30 µg/mL). Under these experimental conditions, BMDM produced IL-10 (Figure 1C), but IL-12 was not detected (data not shown). The IL-10 production was significantly reduced by the previous treatment of macrophages with PAFR antagonists, an anti-CD36 blocking antibody, or a combination of PAFR antagonists with anti-CD36. These data show that oxLDL induces IL-10 and not IL-12, and that a crosstalk or synergistic effect between CD36 and PAFR is required for IL-10 production. This was further confirmed in HEK293T cells transiently transfected with plasmids encoding hPAFR cDNA or hCD36 cDNA, or both, and then stimulated with oxLDL for 5 h. We found that only double transfected cells expressed IL-10 mRNA and trace amounts of IL-12 mRNA. Non-transfected or single transfected HEK293T cells were unable to respond to oxLDL (Figure 1D).

 The effects of oxLDL may vary according to the degree of oxidation. We then assayed the effect of minimally oxidized LDL (moxLDL) on IL-10 and TGFβ production by human macrophage THP-1 cells. The moxLDL was characterized as presenting low values for both negative charges and TBARS compared to oxLDL, as described in methods section. The moxLDL preparation did not induce IL-10 and TGF-β production as did the cells stimulated with oxLDL (Figure 1E-F). 

#### Lipid Raft integrity is important for uptake of FITC-oxLDL and IL-10 production

**Figure 2:**
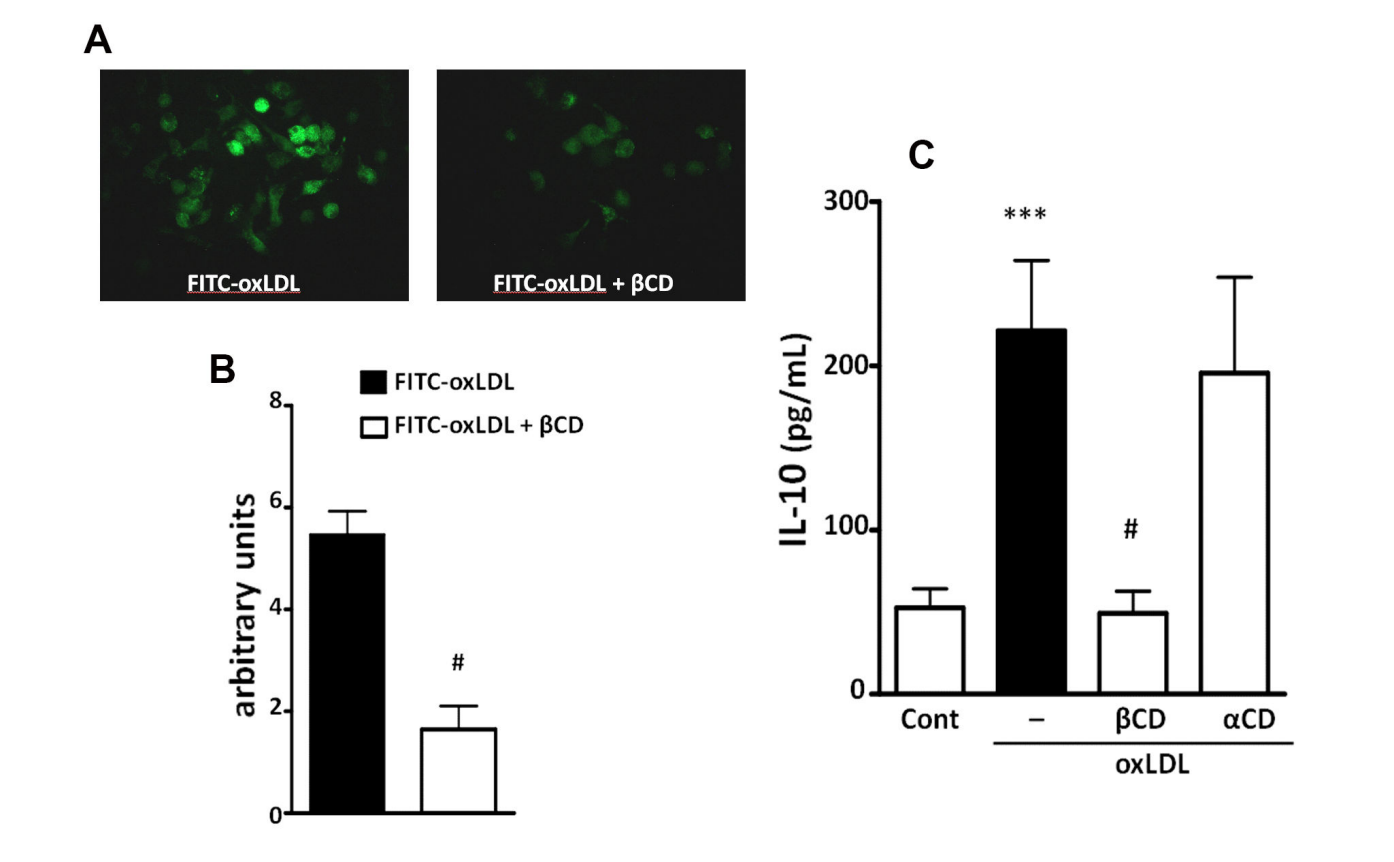
Lipid raft integrity is important for oxLDL uptake and IL-10 production. Macrophages were treated with βCD (2 mmol/L) or with the inactive analog αCD for 5 min. The uptake assay was performed by incubation with FITC-oxLDL (30 µg/mL) for 1 h and visualized by microscopy. Data are presented as representative Figure (A). The fluorescence was quantified by the AlphaEaseFC™ software V3.2 beta (Alpha Innotech) (B). IL-10 production was measured in the supernatants after oxLDL stimulation for 24 h (C). Graph Data are presented as mean ± SEM. ***p<0.001 comparing with non-stimulated cells. #p<0.05 comparing βCD treated with non-treated cells.

Cholesterol-rich microdomains, known as lipid rafts, have been shown to function as specialized platforms for receptor interactions[21]. We, therefore, examined whether lipid raft integrity is required for FITC-oxLDL uptake and the induction of IL-10. Macrophages were treated with methyl-β-cyclodextrin (βCD), a synthetic molecule that sequesters cholesterol from plasma membranes and disrupts lipid rafts. We found that βCD caused a significant reduction (70%) in FITC-oxLDL uptake (Figure 2A and 2B). In Figure 2C it can be seen that βCD treatment markedly inhibited the production of IL-10 induced by oxLDL. The inactive analog αCD did not affect the IL-10 production. These data show that the lipid raft integrity is important for the oxLDL uptake and IL-10 production induced by oxLDL.

 It has been shown that Syk (Spleen tyrosine kinase) may interact with CD36 in the lipid raft fraction, contributing to its association with other receptors and adaptor proteins and allowing it to drive cell activation [22]. To investigate the role of Syk we treated macrophages with a Syk-selective inhibitor, piceatannol (20 µmol/L) or with a general kinase inhibitor genistein (10 µmol/L), 10 min before the FITC-oxLDL uptake assay or oxLDL-induced IL-10 production. We found that piceatannol did not interfere with the oxLDL uptake or IL-10 production (Table 1). In contrast, genistein treatment decreased both FITC-oxLDL uptake and IL-10 production. These results show that oxLDL uptake and IL-10 production is dependent on the activation of kinases other than Syk. The treatments employed in these studies did not affect the cell viability, measured by MTT assay. 

**Table 1:** Syk activation is not required for IL-10 production and oxLDL uptake by macrophages.

	**IL-10 (pg/mL**)	**oxLDL uptake (MFI**)
**Cont**	52.6 ± 11	7.8 ± 1
**oxLDL**	221.1 ± 49 ^***^	97.5 ± 8 ^***^
**oxLDL + Piceat**	354.8 ± 80	104.5 ± 2
**oxLDL + Genist**	43.4 ± 8 ^#^	50.4 ± 17 ^#^

Macrophages were treated with the Syk-selective inhibitor, piceatannol (20 µmol/L), or with a general kinase inhibitor genistein (10 µmol/L), 10 min. For uptake assay, treated cells were incubated with FITC-oxLDL for 1 h and the fluorescence measured by FACS. Data are presented in mean of fluorescence intensity (MFI) For IL-10 production, treated cells were stimulated with oxLDL (30 µg/mL) for 24 h and the cytokine production was evaluated in the supernatants. Data are presented as mean ± SEM of three independent experiments. ***p<0.001 comparing with non-stimulated cells. # p<0.05 comparing cells treated with non-treated cells.

### oxLDL induces colocalization and co-immunoprecipitation of PAFR and CD36

**Figure 3:**
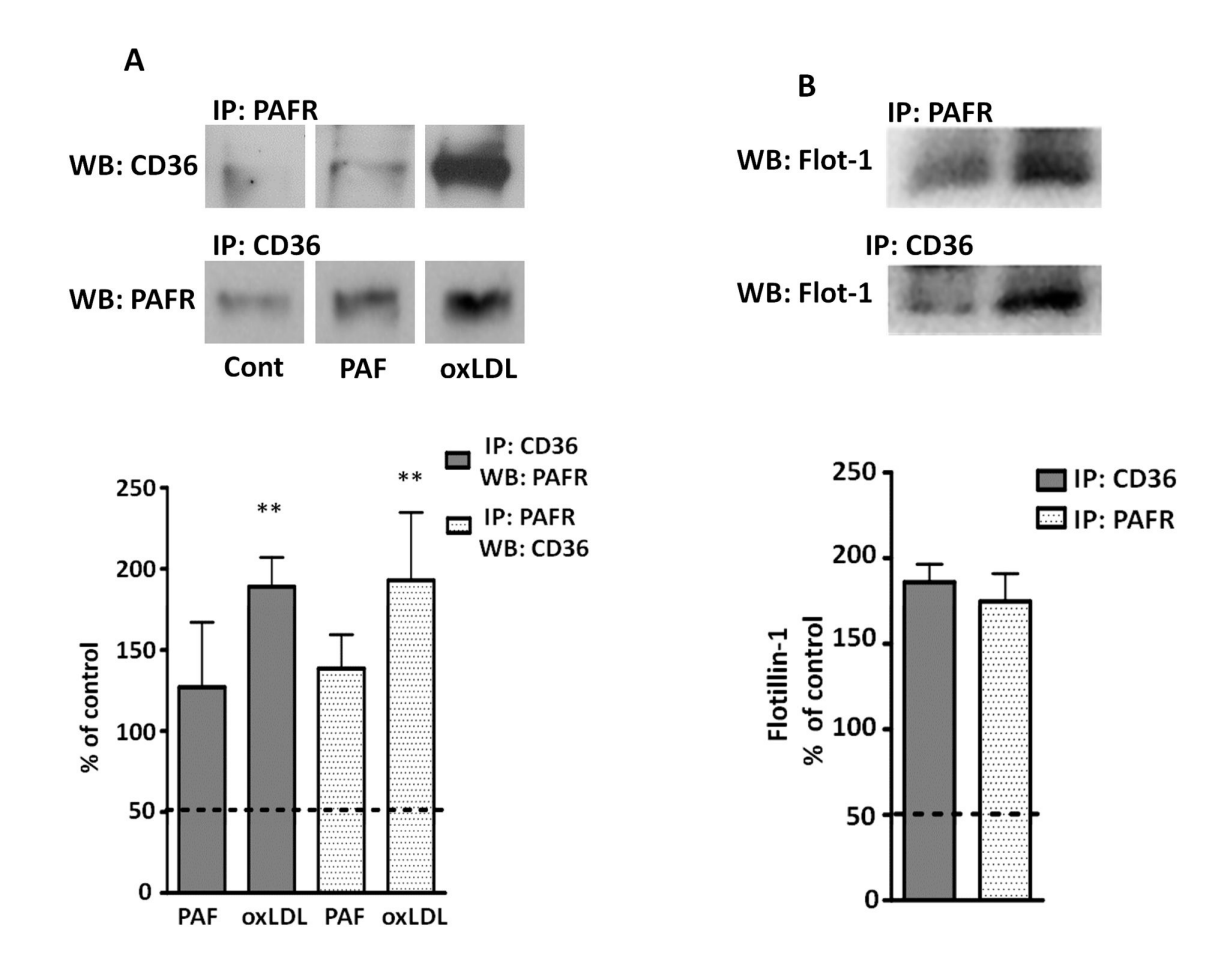
oxLDL induces co-immunoprecipitation of CD36-PAFR-Flotillin-1. Macrophages were treated with oxLDL (30 μg/mL) or PAF (10^-7^ mol/L) for 20 min at 37°C prior addition of the lysis buffer. Cell lysates were subjected to immunoprecipitating and immunoblotting as described in “Methods section” using antibodies to CD36, PAFR (A) and Flotillin-1 (B). Protein expression was quantified by the AlphaEaseFC™ software V3.2 beta (Alpha Innotech). The autoradiographs show one representative experiment and Graph data are presented as mean ± SEM of four experiments. In A, western blot figures were obtained from the same gel. ** p<0.01 comparing oxLDL-stimulated with the non-stimulated cells (dashed lines).

**Figure 4:**
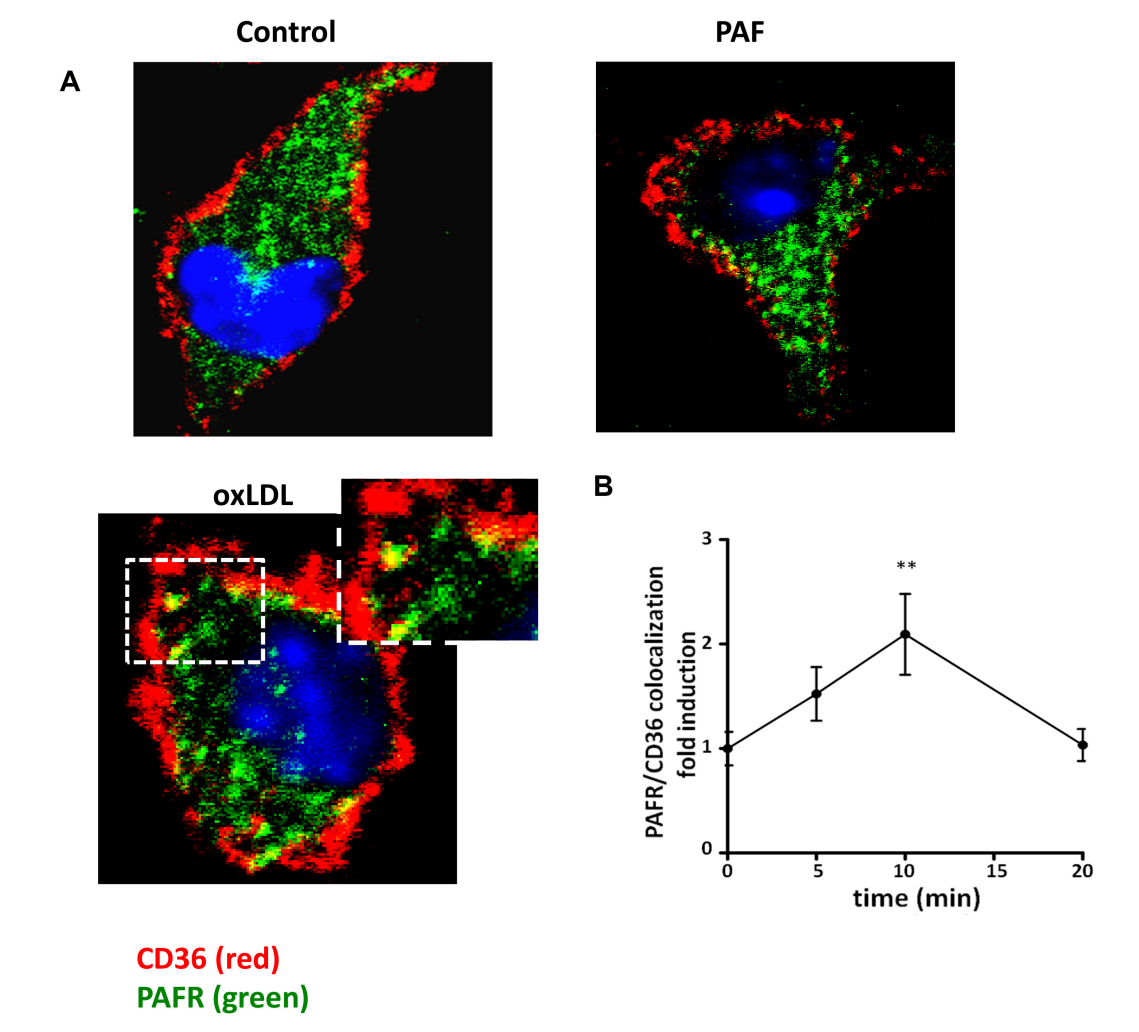
oxLDL induces colocalizaton of CD36 and PAFR. Macrophages stimulated with oxLDL (30 μg/mL) or PAF (10^-7^ mol/L) for 10 min, before staining for PAFR-FITC (green) and CD36-PE (red) and visualized by confocal microcopy (A). Graph data shows the colocalization of PAFR and CD36 in macrophages stimulated with oxLDL for 5, 10 and 20 min (B). Colocalization images were quantified using the package ImageJ 1.44p and Graph data are presented as mean ± SEM of 15 pictures in three independent experiments. ** p<0.01 comparing oxLDL-stimulated with the non-stimulated cells. Images are representative of at least three independent experiments. Yellow patches signify areas of colocalization of CD36 and PAFR.

In order to investigate whether oxLDL induces a complex formation with PAFR and CD36, we performed co-immunoprecipitation assays. Macrophages were stimulated with oxLDL (30 µg/mL) or PAF (10^-7^ mol/L) for 20 min. PAFR and CD36 from the cell lysate were immunoprecipitated as described in the methods section. Figure 3A shows that stimulation with oxLDL rapidly induced receptor interaction, which was detected by the co-immunoprecipitation of PAFR and CD36 (2-fold increase compared to non-stimulated control). In non-stimulated control cells we detected a small proportion of PAFR co-immunoprecipitated with CD36, which was not significantly increased by PAF stimulation. Next, we analyzed the immunoprecipitation of PAFR or CD36 with flotillin-1, a lipid raft associated protein[23]. It can be seen in Figure 3B that oxLDL induced the strong co-immunoprecipitation of flotillin-1with PAFR and CD36 (around 1.8-fold increase in relation to non-stimulated cells). These results strongly suggest that PAFR and CD36 associate in a protein complex induced when oxLDL is added to macrophages and this may occur in the lipid raft platforms. To further define the association of PAFR and CD36, we performed colocalization assays by confocal microscopy. BMDM were stimulated with oxLDL (30 µg/mL) for 5, 10 and 20 min, fixed with 3% paraformaldehyde and labeled with antibodies to CD36 and PAFR. We found that oxLDL stimulation induced a redistribution of PAFR and CD36 in macrophages increasing the colocalization on the macrophage membrane (Figure 4A). This effect was time-dependent, with maximum colocalization seen in 10 min (Figure 4B). In contrast, in PAF stimulated macrophages, only a discrete receptor colocalization was observed. These data indicate that oxLDL induces a spatial redistribution in the plasma membrane, resulting in the recruitment of PAFR and CD36 to the same complex in macrophages. 

### oxLDL induces colocalization of GM1 ganglioside with PAFR and CD36

**Figure 5:**
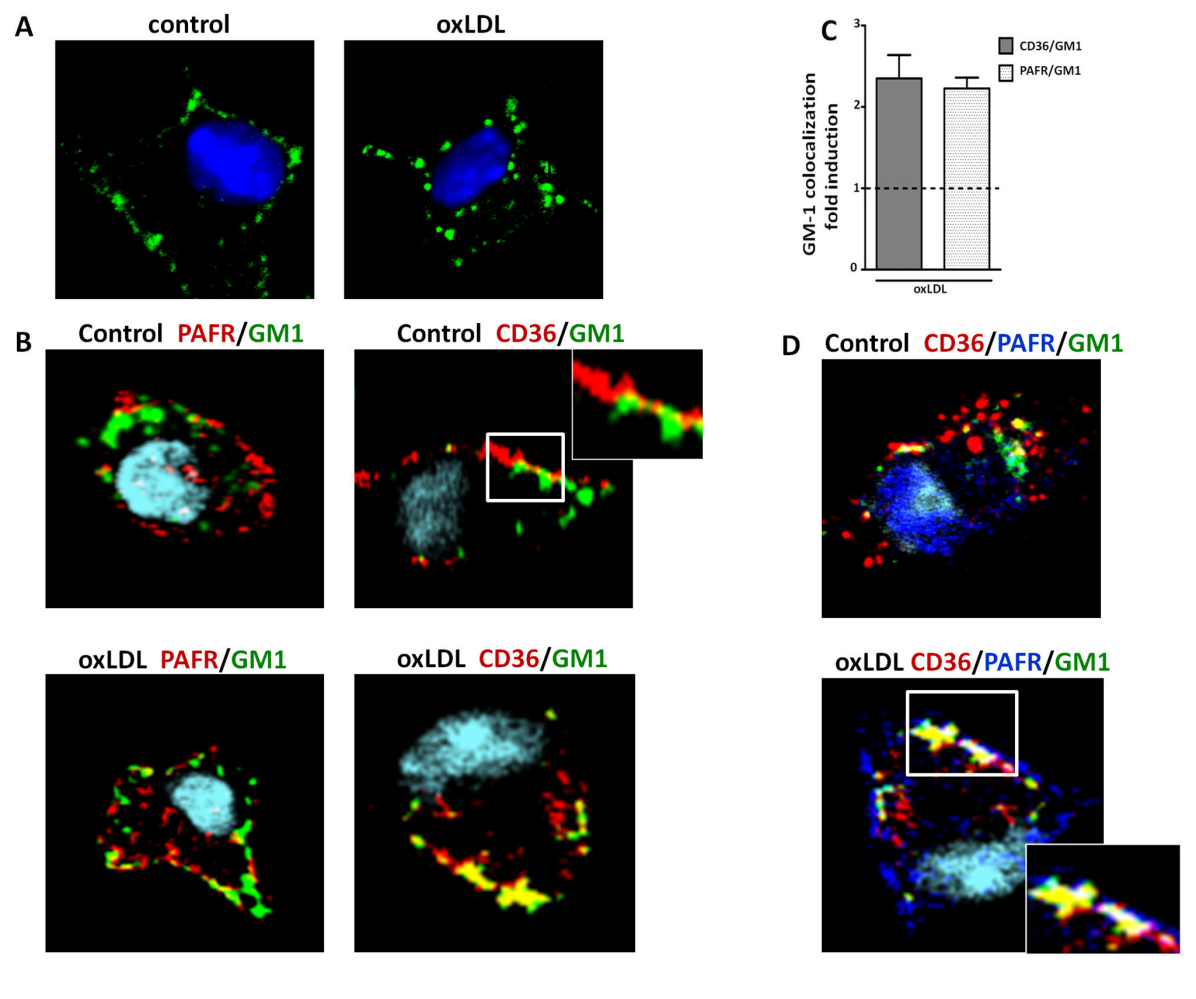
oxLDL induces colocalization of CD36 and PAFR in lipid raft microdomains. Macrophages were stimulated with oxLDL (30 µg/mL) for 10 minutes. Cells were fixed and stained with CTxB-Alexa 488/anti-CTxB, Alexa-647 anti-PAFR, and phycoerythrin anti-CD36, as described in the methods section. Colocalization was visualized by confocal microcopy at a 60-fold magnification. In A-B, macrophages were stimulated with oxLDL and stained for GM1 lipid raft fraction (green) alone or in combination with PAFR-Alexa-647 (red) or CD36-PE (red). Yellow patches signify areas of colocalization of PAFR or CD36 and GM1. In (C), colocalization images were quantified using the package ImageJ 1.44p and Graph data are presented as mean ± SEM of 10-15 pictures in three independent experiments. Dashed lines signify the non-stimulated cells. In (D), macrophages were triple stained for GM1- Alexa 488 (green), PAFR – Alexa 647(blue) and CD36-PE (red). Colocalization areas of triple stained are visualized in white/gray patches.

**Figure 6:**
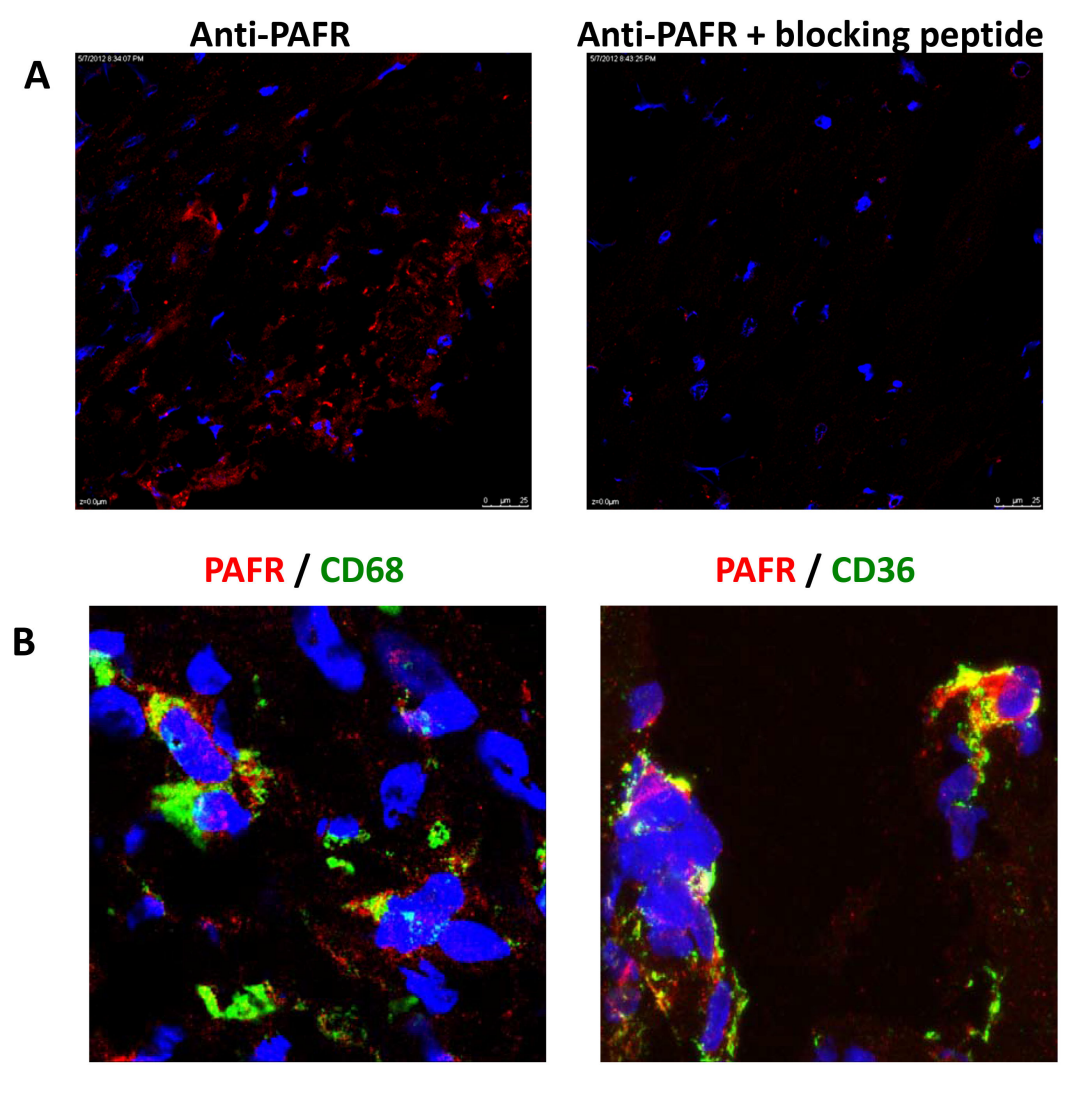
CD36-PAFR complex is present in human carotid plaques. Frozen sections of human carotid plaques were fixed with acetone and stained with rabbit and with the mouse anti-human CD36 or mouse anti-human CD68. Anti-rabbit IgG DyLight-594 or anti-mouse DyLight-488 were used as a secondary antibody. Colocalization was visualized by confocal microcopy at a 60-fold magnification. In (A), the specificity of anti-PAFR was evaluated by pre-treatment with PAF receptor blocking peptide and after stained for hPAFR. In (B) is shown double staining of PAFR with CD36 or with CD68. Yellow patches signify areas of colocalization. Figures in (A) were used as staining control and were acquired in different magnification.

As flotilin-1 has immunoprecipitated with CD36 and PAFR after oxLDL stimulation (Figure 3B), we next investigated whether GM1, another constitutive raft protein used as a raft marker, colocalizes with PAFR and CD36 in macrophages stimulated with oxLDL. GM1 was cross-linked by CTxB, and anti-CTxB antibody detected clustering (patching) of GM1. Utilizing Ctx-FITC staining, GM1 was visualized in a diffuse distribution in resting macrophages and clustering in oxLDL-stimulated macrophages (Figure 5A). Confocal microscopy indicated that a small proportion of CD36 colocalized with GM1 in resting cells, whereas PAFR was not detected in these areas. The oxLDL treatment for 10 minutes increased the colocalization of both PAFR and CD36 with GM1 (Figure 5B and 5C). In experiments using triple staining, we found that oxLDL rapidly induces the recruitment of PAFR and CD36 to the same lipid raft (Figure 6D). These experiments confirm the data obtained with co-immunoprecipation and cholesterol depletion, showing that oxLDL induces the recruitment of PAFR and CD36 to the same lipid raft membrane platforms. 

### Colocalization of PAFR and CD36 in human atherosclerotic plaques

Next, we investigated whether the colocalization of both receptors is present in atherosclerotic lesions. Human carotid atherosclerotic lesions were labeled with antibodies to hPAFR and hCD36. The specificity of the anti-hPAFR was confirmed by competitive assay using the PAF receptor blocking peptide (Figure 6A). Figure 6B shows that PAFR was predominantly colocalized with CD68 positive cells indicating its expression in macrophages. Consistent with results found in mouse macrophages, PAFR colocalized with CD36 in human atherosclerotic plaque (Figure 6B). These results show that PAFR and CD36 act cooperatively in human cells and this may be relevant in atherosclerosis development.

## Discussion

Here we describe that PAFR and CD36 are recruited to the same complex and this is required for an optimal oxLDL uptake and IL-10 production induced by oxLDL in macrophages. This was confirmed by transfection experiments where oxLDL induced the expression of IL-10 mRNA only in HEK293T expressing both receptors. The disruption of lipid rafts by treatment with mβCD reduced IL-10 production. The uptake of oxLDL was increased when both receptors were present. The interaction between these receptors seems to require lipid rafts formation. Finally, we observed that PAFR and CD36 are colocalized in human atherosclerotic plaques. 

In a previous work we found that PAFR-antagonists reduce oxLDL uptake and that PAFR deficient mice take up less oxLDL than their littermate controls[10]. Thus, we already suspected that CD36 and PAFR interact upon macrophage exposure to oxLDL. Here, we show that PAFR antagonists and mAb to CD36 alone or in combination reduced the oxLDL uptake. Results were consistent using two different and molecularly unrelated PAFR antagonists that induced similar inhibition. 

 CD36 is considered a pattern recognition receptor that binds to negatively charged ligands from both pathogens and oxidized/damaged self-components[24,25]. This receptor was shown to interact with TLR2-TLR6 in macrophages exposed to *S. aureus*-derived lipoteicoic acid (LTR)[26] and also with TLR4-TLR6 in cells stimulated with amyloid-β protein[13]. It is known that CD36 recognizes oxidized phospholipids and apoptotic cells[25]. We have shown that PAFR also recognizes apoptotic cells and oxLDL and suggested that these receptors interact somehow in macrophages membrane for optimal oxLDL uptake[10,27]. 

According the oxidative stage, the LDL might have similar or different effects on cell activation. A minimal oxidation degree of LDL is characterized by antioxidant depletion, oxidation of arachidonic acid-containing phospholipids, relatively low linoleic acid oxidation and insignificant protein modifications [28]. However, in highly oxidized LDL, phospholipids, triacylglycerol and cholesterol esters are transformed into hydroperoxides which react with ApoB-100, resulting in modification and fragmentation of amino acid side chains [29,30]. Here, we found that only the LDL with a high degree of oxidation increased IL-10 and TGF-β production in human THP-1 cells. However, in previous study we showed that LDL with low and high oxidation both increase the expression of CD36 and may both contribute to foam cell formation [31]. Recognition of oxLDL by murine macrophage receptors induces the production of IL-10 but not IL-12. Our results show that both CD36 and PAFR are involved in IL-10 production. Although several receptors can be involved in oxLDL recognition [32], the oxLDL-induced IL-10 production depends mainly on CD36 and PAFR since, in the present study, the production of this cytokine was almost completely blocked by treatment with PAFR antagonists and mAb to CD36. Our results show that IL-10 production by BMDM is dependent on PAFR activation corroborating previous results reported by Verouti et al, 2011[33] . These authors showed that oxLDL induced MCP-1 production partly through PAFR activation and that this occurred via protein kinases activation. Our previous results are also in accordance with these authors, since we showed that oxLDL-induced IL-8 production by human monocytes-macrophages was dependent on MAPK and PI3K/AKT pathways activation [11]. It was also reported that the activation of PAFR is essential for oxLDL-induced recruitment of human bone marrow-derived mesenchymal stem cells dependent on MAPK activation [34].

 IL-10 is an anti-inflammatory cytokine that was found to be expressed by macrophages from atherosclerosclerotic plaques[35]. This cytokine is a marker of alternatively activated macrophages which are involved in repair mechanisms with fibroblasts activation and collagen production[35]. IL-10 was shown to decreases CD36 mRNA expression and increases the cholesterol efflux in macrophages[36]. Other studies have shown that IL-10 induces lipid accumulation in macrophages and may contribute to the foam cell formation[37]. However, its role in atherosclerosis still remains to be determined. 

 Here, we demonstrate that the lipid rafts disruption decreases the uptake of oxLDL and IL-10 production by macrophages. Lipid rafts are cholesterol-rich and sphingomyelin-rich membrane domains functioning as scaffold platforms for the association of signaling molecules and compartmentalization of cellular processes. It has also been shown that lipid raft formation is involved in cell activation induced by oxidized lipids[38], production of pro-inflammatory cytokines[39] and uptake of acetyl LDL[40]. The following results indicate that the interaction of CD36 and PAFR occurs within lipid raft domains of the BMDM membrane: a) Disruption of lipid rafts by treatment with MβCD reduced the oxLDL uptake and IL-10 production; b) oxLDL induced co-immunoprecipitation of PAFR and CD36 with the constitutive raft protein, flotillin-1[23] and colocalization of PAFR and CD36 with the lipid raft marker GM1-ganglioside[41]. Data presented by others have been shown that CD36 is recruited to lipid rafts in a ligand-dependent manner[22,42]. Although PAFR is a GPCR and might interact within lipid raft platforms[43], there is only one study showing that PAFR migrates to lipid rafts, which was observed in cells transfected with PAFR and stimulated with PAF[15]. In a previous study we showed that both receptors, PAFR and CD36, are able to bind the oxLDL. However, only the co-stimulation of CD36 and PAFR by oxLDL was able to transduce intracellular signaling for cytokine production [11]. Although our data clearly suggest a crosstalk between receptors for PAF and CD36, they do not show at what level the interaction among these receptors occurs. We show here that one possibility is that oxLDL recruits both receptors to specific membrane microdomains (lipid rafts), allowing association between these two receptors. However, we cannot exclude the possibility that they signal in parallel, interacting downstream or that other receptors that are recruited to the same microenvironment also contribute. 

 The signaling elicited by CD36 engagement leads to the recruitment of the adaptor protein Syk, which was shown to contribute to receptors association[22]. In our study, Syk inhibition did not affect oxLDL uptake or IL-10 production, in contrast to genistein, a general inhibitor of kinases, which reduced both events. This indicates that Syk is not involved in CD36-PAFR interaction induced by oxLDL. It has been shown that Syk phosphorylation requires macrophage activation induced by LDL with minimal modification[44]. It is likely that oxLDL will induce distinct effects depending on the degree of oxidation. Indeed, previous data showed that LDL with high or low degrees of oxidation has different effects on macrophage[31].

The CD36 and PAFR complex formation in human atherosclerotic plaques is intriguing. It can be speculated that these receptors association would contribute not only to increased foam cell formation but also contributes to chronic inflammatory response in the atherosclerotic plaque. However, we have no clues as to how this affects the progression of atherosclerosis. Although the role of PAF/CD36 complex formation in atherosclerotic plaques remains to be determined, this study increases our understanding of macrophage interactions with oxLDL and provides new insights into atherosclerosis research.
